# Microbiomes of the Enteropneust, *Saccoglossus bromophenolosus*, and Associated Marine Intertidal Sediments of Cod Cove, Maine

**DOI:** 10.3389/fmicb.2018.03066

**Published:** 2018-12-14

**Authors:** Gary M. King

**Affiliations:** Department of Biological Sciences, Louisiana State University, Baton Rouge, LA, United States

**Keywords:** enteropneust, sediment, diversity, intertidal, bacteria

## Abstract

Enteropneusts are widely distributed marine invertebrates that accumulate high concentrations of halogenated aromatics. Some of these compounds affect benthic biogeochemistery (e.g., denitrification and ammonia oxidation), but little is known about interactions between enteropneusts and their associated microbial communities. Even less is known about enteropneust host-microbe relationships in the digestive tract. More generally, microbial community composition and diversity in intertidal sediments have received little attention. In this study, high throughput sequence analyses of 16S rRNA genes extracted from microbial communities associated with sediment-free whole individuals of *Saccoglossus bromophenolosus* and freshly excreted *S*. *bromophenolosus* gut sediments revealed a potential Spirochaete symbiont that was abundant, present in gut sediment, but absent in other sediments. Relative to surface sediments, gut communities also revealed evidence for selective losses of some groups and blooms of others, especially *Colwellia*, *Photobacterium*, *Pseudoalteromonas*, and *Vibrio*. After deposition, gut sediment communities rapidly resembled those of surface sediments. Although hierarchical cluster analysis and Linear Discriminant Analysis Effect Size (LEfSe) differentiated among burrow walls of *S*. *bromophenolosus* and a polychaete, *Alitta virens*, as well as between surface and sub-surface sediments, most operational taxonomic units (OTUs) were shared, with differences largely occurring in relative abundances. This suggests that sediment mixing through bioturbation might act to homogenize community composition, while species-specific impacts by infauna might alter local population abundances. Although Cod Cove is a relatively isolated intertidal system, microbial community members included groups with cosmopolitan distributions and roles in sulfur cycling, e.g., Gammaproteobacteria BD7 and Sva0071, as well as novel OTUs representing a large number of phyla.

## Introduction

Enteropneusts (phylum Hemichordata) are exclusively marine, worm-like, benthic deposit feeders that burrow in sediments from the intertidal zone to the deep-sea, and from tropical to polar latitudes (King et al., 1994, 1995; Giray and King, 1996). Most enteropneusts spend a portion of their time feeding at the sediment-water interface (Gonzalez and Cameron, 2009). They also create sub-surface interfaces, i.e., burrows (King, 1986; Dobbs and Guckert, 1988), which facilitate physical, chemical and biological interactions among animals and their environments (e.g., Hansen et al., 1996; Blondin-Mermillod et al., 2004; Kristensen and Kostka, 2005; Satoh et al., 2007; Bertics and Ziebis, 2009; Bertics et al., 2010; Satoh and Okabe, 2013). Biological interactions include the potential for altering microbial communities in material that is ingested, digested within the gut, and subsequently re-deposited at the sediment surface as fecal castings. Microbial communities in burrow wall sediments are also subject to change through burrow irrigation, which affects oxygen availability; mucus secretion, which provides a source of organic matter; and grain size sorting, which can affect porosity and diffusivity (Kristensen and Kostka, 2005).

Enteropneusts contain high concentrations of halogenated organics (haloorganics), e.g., bromophenols, bromo- and chloroindoles, and bromoquinones among others, which also affect microbial communities (e.g., King, 1986). Whether they synthesize these compounds themselves as some marine algae do (Cabrita et al., 2010), or acquire them from microbial symbionts as marine sponges do (Grozdanov and Hentschel, 2007; Bayer et al., 2013), remains unknown. Halogenase genes and halogenation activity have been documented for Actinobacteria and Firmicutes isolated from an enteropneust, *Ptyochodera bahamensis* (Lilles, 2011), but the possibility that enteropneusts harbor haloorganic-producing symbionts has not been explored.

The functional roles for enteropneust haloorganics are also uncertain. They include predation deterrence and bioluminescence (Giray and King, 1997b; Kanakubo and Isobe, 2005). Other potentially important functions include mediating animal-microbe interactions on the enteropneust epidermis or in burrow sediments. In support of the latter, previous studies have shown that 2,4-dibromophenol (DBP), which is accumulated by *S. bromophenolosus* (King, 1986), inhibits aerobic respiration in sediment slurries, and also might account for reduced bacterial numbers and rates of denitrification and ammonia oxidation in enteropneust burrow wall sediments (Giray and King, 1997a). Ammonia oxidation appears especially sensitive to DBP inhibition, in contrast to sulfate reduction, which was notably insensitive (Giray and King, 1997a).

Although haloorganics can have strong and selective inhibitory effects on the activity of specific groups of bacteria, the extent to which they might structure microbial communities remains uncertain. Phospholipid fatty acid (PFLA) profiles have been used to compare burrow communities associated with a haloorganic-free polychaete (*Branchyoasychus americana*) and two haloorganic-containing taxa, an enteropneust (*Balanoglossus aurantiacus*), and a polychaete (*Notomastus lobatus*). Differences among burrows were observed for several broad lipid classes, e.g., monounsaturated PLFA, while markers for specific groups, e.g., sulfidogens and Gram-negative bacteria, did not vary (Steward et al., 1996). The similarity of sulfidogens among burrows might reflect insensitivity to haloorganics (Giray and King, 1997a), but since PLFA lack the resolution necessary for elucidating patterns at family to genus levels, it is unclear what similarities in lipid markers actually imply.

To identify potential impacts of enteropneusts on benthic microbial communities, partial 16S rRNA gene sequences were amplified from extracts of sediments associated with *S*. *bromophenolosus* and a haloorganic-free polychaete, *Alitta* (*Nereis*) *virens*. Amplicon sequences were obtained using an Illumina Miseq platform from gut sediments freshly excreted by *S*. *bromophenolosus*, from its fecal castings at the sediment surface, and from whole, sediment-free animals. In addition, amplicon sequences were obtained from burrow wall sediments of *S*. *bromophenolosus* and *A*. *virens*, and from bulk surface and sub-surface sediments. Phylogenetic analyses revealed distinct differences in the compositions of whole *S*. *bromophenolosus* and gut sediment, each of which differed from burrow wall, and bulk and sub-surface sediments; compositions of the latter sediment types also varied, but less distinctly.

## Materials and Methods

### Site Description and Sample Collection

Animal and sediment samples were collected in May 2014 and August 2016 from Cod Cove, Maine (approximate GPS coordinates 44.000954, -69.639964) during low tide at a site near shore. Approximately 2–3 gram fresh weight (gfw) samples of surface sediment (*n* = 5) were collected from the upper 2–3 mm interval using a sterile spatula. This interval was visually distinct (light brown, oxidized) from darker, reduced sub-surface sediment. *S. bromophenolosus* fecal castings (*n* = 5) on the sediment surface were identified by their characteristic coiled structure. Well-defined coils indicative of deposition for < 12 h were collected with a spatula while avoiding surrounding surface sediment; the mass of each replicate sample was about 0.5 gfw. To collect sub-surface (*n* = 4) and burrow wall sediment as well as animals, a round-point garden shovel was used to create a crack approximately the width and depth of the shovel blade. Bulk sub-surface sediment not obviously associated with burrows was collected from a depth of about 5 cm relative to the surface using a sterile spatula as above. Spatulas were also used to collect the inner 1–2 mm of burrow wall sediments from burrows containing *Alitta virens* (*n* = 5), and from burrows formed by *S. bromophenolosus* (*n* = 6); the latter were readily identified by their distinctive iron oxide deposits. In general, sample collection followed methods used previously for animals and sediments in nearby Lowes Cove, Maine (King, 1986; Giray and King, 1997a,b). Immediately after collection, all samples were transferred to sterile 15 ml disposable conical centrifuge tubes containing up to 5 ml Lifeguard solution as a DNA preservative (QIAGEN, Inc.; Germantown, MD, United States).

Four individual specimens of *S. bromophenolosus* were carefully removed from their burrows with a spatula preserving as much of the fragile trunk as possible. Individuals collected in 2014 were placed in ethanol-sterilized polystyrene weighing dishes (8.9 cm × 8.9 cm × 2.5 cm depth length-width-depth) containing about 20 ml of 0.2 μm filter-sterilized seawater. Sediment contained in the gut was collected after it was voided, and transferred to disposable centrifuge tubes with Lifeguard solution as above. Seven individuals collected in 2016 were treated similarly, with the exception that whole, sediment-free animals were transferred to centrifuge tubes with Lifeguard.

### DNA Extraction and Sequence Analysis

DNA was extracted using a Qiagen Powersoil kit (QIAGEN, Germantown, MD, United States) following the manufacturer’s protocol after removing Lifeguard. Whole animals collected in 2016 were homogenized first, then sub-samples of the homogenate were transferred to bead-beating tubes for extraction. Purified DNA extracts were submitted to the Research Technology Support Facility of Michigan State University for PCR amplification and barcoding of the 515–806 (V4–V5) region of the 16S rRNA gene according to Kozich et al. (2013). The sample libraries were sequenced on a Miseq platform using a v2 reagent cartridge for a 2 × 250 bp paired-end format. Sequencing yielded a total of 6,273,492 reads. Sequences have been deposited in the NCBI SRA archive as Bioprojects PRJNA480512 (*Saccoglossus bromophenolosus* microbiome) and PRJNA480505 (Intertidal and enteropneust microbiome).

The resulting sequences were processed using a mothur pipeline (v 39.5) to filter reads for quality, create contigs and reduce noise (Kozich et al., 2013). Sequences were aligned with SILVA database release 128 (Quast et al., 2013), and the SILVA taxonomy was used for classification of representative sequences and operational taxonomic units (OTUs), which were defined at an evolutionary distance of 0.03 using mothur’s cluster.split algorithm with the “opticlust” option and “taxlevel” set at 4 for splitting the distance matrix. Chimeras were identified and removed with the chimera.uchime option. Alpha diversity (e.g., Chao1, Shannon and inverse Simpson indices, and coverage) for individual samples was estimated using mothur and MicrobiomeAnalyst (Dhariwal et al., 2017)^1^ with normalized read abundances excluding singletons. Samples analyzed with MicrobiomAnalyst were filtered for low abundance based on the mean abundance of OTUs, and for low variability using the inter-quantile range assessment. After filtering, OTU abundances were transformed using the centered log ratio. The significance of differences in alpha diversity among sample groups was tested using analysis of variance (ANOVA) with a Bonferroni *post hoc* test and correction of *p*-values for multiple comparisons. Both platforms were also used to generate beta diversity indices and to visualize community (dis)similarities using principle coordinates analyses (PCoA) and non-metric multidimensional scaling (nMDS) of Bray-Curtis, unweighted Unifrac, and weighted Unifrac distance matrices [with tests of significance using permuted analysis of variance (PERMANOVA), permuted analysis of dispersion (PERMDISP), and analysis of similarity (ANOSIM)].

## Results and Discussion

### Surface, Sub-Surface, Burrow Wall, and Fecal Cast Sediment Communities

**FIGURE 1:**
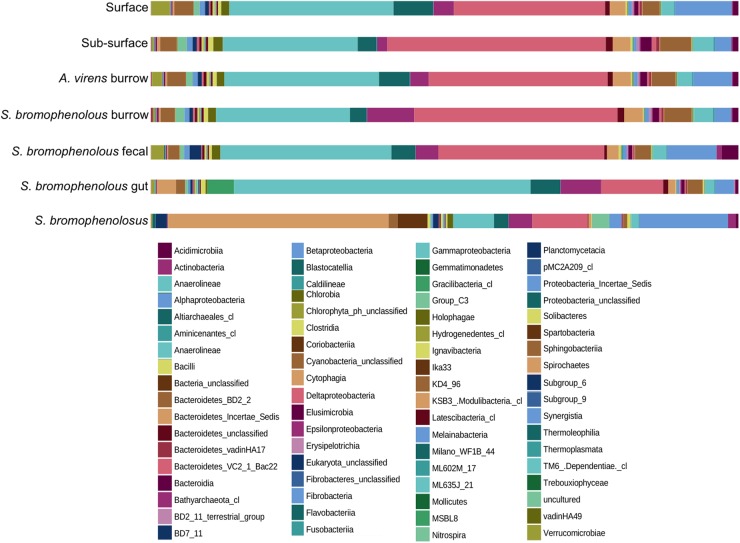
Percent composition of animal and sediment microbial communities. Percent composition by class of microbial communities in surface and sub-surface sediments, burrows of *Alitta virens*, burrows of *Saccoglossus bromophenolosus*, *S*. *bromophenolosus* fecal castings, *S*. *bromophenolosus* gut sediments, and whole, sediment-free *S*. *bromophenolosus*. Delta- and Gammaproteobacteria dominate sediment samples, while Gammaproteobacteria and Spirochaetes dominate *S*. *bromophenolosus* gut sediments, and whole, sediment-free *S*. *bromophenolosus*, respectively.

Although 107 classes were identified among the various sediment communities, most were rare ( < < 1%), with just 14 accounting for 81.3–86.4% of total OTU abundance (Figure 1 and Table 1). The latter included various members of the Acidobacteria, Bacteriodetes, Chloroflexi, Planctomycetes, Proteobacteria, and Spirochaetes. In general, patterns observed for the most abundant OTUs in Cod Cove sediments were similar to those reported for intertidal sediments by Gobet et al. (2011) and Zheng et al. (2014). Similar results were also reported by Zinger et al. (2011), who synthesized results from a global ocean dataset. However, lower abundances of Actinobacteria, Betaproteobacteria and Firmicutes in Cod Cove were notable. These differences might reflect more general patterns for intertidal sediments, but additional studies will be necessary to verify them.

**Table 1:** Major classes as a percentage of surface, sub-surface (Sub), *Alitta virens* burrows (*A*. *v*.), *Saccoglossus bromophenolosus* burrows (*S*. *b*.), *S. bromophenolosus* fecal castings (Fecal), *S. bromophenolosus* gut contents (gut), and sediment-free whole *S. bromophenolosus* (Whole) microbial communities; data are means for each sample type.

Classes	Surface	Sub	*A. v.*	*S. b.*	Fecal	Gut	Whole
Alphaproteobacteria	9.44	2.57	6.38	2.71	8.17	3.27	13.49
Anaerolineae	1.94	2.96	2.23	2.88	1.99	1.53	1.15
Bacteroidetes_BD2_2	2.86	4.95	3.87	4.60	2.46	2.36	0.73
Bacteroidia	0.68	1.71	1.14	1.10	0.61	0.54	0.09
Betaproteobacteria	0.45	0.30	0.51	0.56	0.38	0.40	1.80
Chloroplast	2.74	4.83	3.86	2.49	5.67	1.03	5.43
Cytophagia	2.42	2.90	2.86	3.05	1.85	1.26	0.47
Deltaproteobacteria	25.04	35.34	29.25	33.65	27.26	10.96	7.49
Epsilonproteobacteria	3.48	1.65	3.12	8.04	3.81	7.23	4.33
Gammaproteobacteria	27.62	22.15	25.81	22.57	28.67	52.67	7.07
Holophagae	1.29	1.45	1.17	1.22	1.29	0.28	0.93
Planctomycetacia	0.65	0.66	0.68	0.56	1.98	0.34	0.94
Sphingobacteriia	3.16	2.72	3.06	2.39	1.83	1.60	1.46
Spirochaetes	0.29	0.49	0.29	0.33	0.17	3.32	35.99
Other	17.96	15.31	15.76	13.84	13.86	13.21	18.64
Total proteobacteria	67.17	63.07	66.25	68.85	69.35	74.92	34.55
Total bacteroidetes	14.40	14.69	14.47	12.37	9.61	10.92	4.94


Proteobacteria dominated all Cod Cove sediments, accounting for 63.1–69.4% of the OTU abundances (Table 1). Similar results have been obtained for other coastal sediments (e.g., Lasher et al., 2009), but surprisingly few studies have addressed unvegetated, unpolluted intertidal mud flats (Bong-Soo et al., 2004; Musat et al., 2006; Gobet et al., 2011; Wang et al., 2012, 2016; Zheng et al., 2014). Alphaproteobacteria and Epsilonproteobacteria occurred at abundances from 2.6 to 9.4%, and 1.6 to 8.0%, respectively (Table 1), consistent with other sediment studies (e.g., Gobet et al., 2011; Wang et al., 2012). Deltaproteobacteria abundances were even higher, and typically comparable to or greater than those for Gammaproteobacteria (25.0–35.3% and 22.2–28.7%, respectively, Table 1) in all but freshly excreted gut sediments (Figure 1 and Table 1).

### Deltaproteobacteria Distribution

Deltaproteobacteria were dominated by sulfidogens as expected for marine sediment (Lasher et al., 2009), but members of the facultatively anaerobic class, Myxococcales, and aerobic family, Bacterivoraceae, collectively accounted for 1.79–2.01% of all OTUs (Table 2). Myxococcales have been previously reported in intertidal sediments populated by a polychaete, *Hediste diversicolor*; greatest abundances occurred in burrow walls, with lower abundances in surface and sub-surface sediments (Pischedda et al., 2011). In contrast, Myxococcales abundances were similar in all Cod Cove sediments, with the exception of freshly excreted gut sediments. In spite of their abundance, the role this group plays in marine sediments is largely unknown other than what might be inferred from their heterotrophic lifestyle (e.g., Li et al., 2012).

**Table 2:** Major contributors to Deltaproteobacteria in surface, sub-surface, *Alitta virens* (*A*. *v*.) burrows, *Saccoglossus bromophenolosus* (*S*. *b*.) burrows, *S. bromophenolosus* fecal casts, *S. bromophenolosus* gut contents, and sediment-free whole *S. bromophenolosus* (Whole) microbial communities.

Taxonomy	Surface	Sub-surface	*A*. *v.* burrow	*S. b.* burrow	*S. b.* fecal	*S. b.* gut	Whole
Myxococcales	1.56 (0.11)	1.78 (0.19)	1.71 (0.14)	1.72 (0.14)	1.79 (0.24)	0.44 (0.16)	4.08 (0.64)
Bacterivoraceae	0.18 (0.04)	0.10 (0.02)	0.20 (0.02)	0.10 (0.02)	0.18 (0.05)	0.30 (0.11)	0.04 (0.01)
*Bdellovibrio*	0.05 (0.01)	0.03 (0.0)	0.05 (0.0)	0.05 (0.01)	0.05 (0.01)	0.05 (0.02)	0.29 (0.06)
*Desulfatiglans*	0.97 (0.33)	1.31 (0.15)	1.07 (0.12)	1.39 (0.13)	0.71 (0.14)	0.86 (0.26)	0.06 (0.03)
Unclassified Desulfobacteria	1.11 (0.31)	1.77 (0.18)	1.06 (0.07)	1.38 (0.08)	0.92 (0.16)	0.52 (0.21)	0.12 (0.07)
*Desulfococcus*	0.10 (0.02)	0.14 (0.01)	0.12 (0.01)	0.11 (0.01)	0.09 (0.02)	0.04 (0.02)	0.01 (0.01)
*Desulfosarcina*	0.82 (0.18)	1.26 (0.11)	1.01 (0.07)	1.07 (0.09)	0.94 (0.17)	0.47 (0.24)	0.06 (0.03)
Unclassified *Desulfobulbus*	1.98 (0.18)	3.28 (0.81)	2.19 (0.17)	2.04 (0.13)	3.05 (0.2)	0.54 (0.25)	0.27 (0.12)
*Desulfobulbus*	6.69 (0.64)	10.4 (0.86)	9.86 (0.82)	13.81 (1.09)	6.95 (1.85)	1.64 (0.82)	0.59 (0.25)
Deltaproteobacteria Sva0485	0.76 (0.09)	1.16 (0.1)	0.98 (0.06)	1.04 (0.09)	0.73 (0.13)	0.35 (0.13)	0.07 (0.03)
Deltaproteobacteria Sva0081	3.08 (0.65)	4.7 (0.46)	3.48 (0.29)	4.05 (0.29)	4.25 (0.68)	1.39 (0.48)	0.52 (0.24)
Desulfuromonadales Sval033	2.94 (0.19)	2.8 (0.39)	2.54 (0.14)	1.78 (0.23)	2.48 (0.54)	0.34 (0.25)	0.13 (0.05)
Desulfurellaceae	0.02 (0.0)	0.01 (0.0)	0.01 (0.0)	0.01 (0.0)	0.01 (0.0)	0.01 (0.0)	0.97 (0.09)
*Geobacter*	0.23 (0.06)	0.03 (0.01)	0.18 (0.03)	0.10 (0.02)	0.18 (0.05)	0.03 (0.01)	1.79 (0.22)


*Data are means ± 1 standard error.*

Among the sulfidogens, *Desulfobulbus* was by far the most abundant genus, accounting for up to 13.8% of OTUs in *S*. *bromophenolosus* burrow sediments (Table 2). *Desulfobulbus* also dominates sulfidogen communities in other marine sediments, although reports of its distribution in animal burrows are limited (e.g., Zheng et al., 2014). Relatively high abundances in *S*. *bromophenolosus* burrows suggest that *Desulfobulbus* is likely unaffected by bromophenols that occur there (King, 1986); whether it can metabolize these compounds or dehalogenate more generally is unknown.

Excluding gut sediments, the distribution of other sulfidogen lineages among sediment types exhibited only minimal differences (Table 2 and Supplementary Figure 1), a finding that supports conclusions of Steward et al. (1996) based on analyses of signature PFLAs, and of King (1988) based on patterns of 2,4-dibromophenol degradation by sediment slurries. In this context it is also noteworthy that OTUs representing sulfidogen lineages known to dehalogenate, e.g., *Desulfitobacterium*, *Desulfomonile*, and *Desulfovibrio* (e.g., Boyle et al., 1999; Knight et al., 1999) were relatively rare and distributed evenly among sediment types. Similar results were found for *Propionigenium* OTUs (Fusobacteria). The later OTUs are of interest, because a bromophenol-degrading *Propionigenium maris* isolate was obtained from burrows of a bromophenol-producing polychaete (Watson et al., 2000). Nonetheless, bromophenol excretion by *S*. *bromophenolosus* does not appear to lead to significant enrichments of bromophenol degraders.

### Gammaproteobacteria Distribution

The Gammaproteobacteria were comprised of several common, widely distributed lineages, including *Thiogranum* (an obligately chemolithoautotrophic sulfur oxidizer, e.g., Mori et al., 2015), *Halioglobus* and *Haliea* (planktonic heterotrophs, e.g., Urios et al., 2008; Park et al., 2012). However, uncultured, unclassified, or relatively rare taxa collectively accounted for 89.1% of the observed richness (S_obs_), 64.0% of the abundance of the Gammaproteobacteria overall, and 9.5–12.2% of total OTU abundance in the various sediment samples (Table 3). Although biogeochemical functions cannot be attributed to many of these OTUs, others have been directly or indirectly implicated in sulfur/sulfide oxidation, e.g., the BD7 group (Dyksma et al., 2016), or oligotrophic carbon metabolism, e.g., members of the KI89A and OM182 clades (Cho and Giovannoni, 2004).

**Table 3:** Major contributors to Gammaproteobacteria in surface, sub-surface, *Alitta virens* (*A*. *v*.) burrows, *Saccoglossus bromophenolosus* (*S*. *b*.) burrows, *S. bromophenolosus* fecal casts, *S. bromophenolosus* gut contents, and sediment-free whole *S. bromophenolosus* (Whole) microbial communities.

Taxonomy	Surface	Sub-surface	*A*. *v.* burrow	*S. b.* burrow	*S. b.* fecal	*S. b.* gut	Whole
*Colwellia*	0.01 (0.0)	0.01 (0.0)	0.03 (0.01)	0.05 (0.02)	0.02 (0.01)	2.83 (0.48)	0.0 (0.0)
Pseudoalteromonadaceae	0.0 (0.0)	0.0 (0.0)	0.0 (0.0)	0.0 (0.0)	0.0 (0.0)	20.17 (7.18)	0.0 (0.0)
Halieaceae	6.05 (0.84)	3.09 (0.22)	4.81 (0.39)	3.20 (0.25)	6.27 (1.21)	0.97 (0.41)	0.47 (0.18)
Chromatiales	2.94 (0.16)	2.62 (0.16)	2.75 (0.09)	2.78 (0.20)	3.60 (0.30)	0.64 (0.19)	0.24 (0.10)
**γ** -Proteobacteria HOC36	0.61 (0.11)	1.16 (0.12)	0.61 (0.04)	0.81 (0.14)	2.05 (0.94)	0.10 (0.03)	0.21 (0.08)
Pseudomonadales	0.05 (0.01)	0.01 (0.0)	0.02 (0.01)	0.0 (0.0)	0.04 (0.01)	3.73 (0.95)	0.03 (0.01)
**γ** -Proteobacteria Sva0072	1.66 (0.10)	1.47 (0.19)	1.83 (0.11)	1.82 (0.11)	2.06 (0.37)	0.49 (0.14)	0.26 (0.10)
Thiotrichales	1.32 (0.26)	1.58 (0.17)	1.77 (0.18)	1.84 (0.14)	1.55 (0.31)	0.97 (0.24)	0.08 (0.03)
Vibrionaceae	0.02 (0.0)	0.01 (0.0)	0.01 (0.0)	0.01 (0.0)	0.01 (0.0)	20.89 (5.42)	0.01 (0.01)
**γ** -Proteobacteria JTB255_MBG	2.08 (0.16)	1.81 (0.18)	2.12 (0.05)	1.83 (0.12)	1.59 (0.30)	0.63 (0.34)	0.20 (0.07)
Other	12.16 (0.81)	9.54 (0.28)	11.11 (0.31)	9.64 (0.29)	11.63 (0.73)	7.67 (0.76)	3.93 (0.23)


*Data are means ± 1 standard error.*

In addition to these clades, several other distinct Gammaproteobacteria groups also accounted for significant fractions of total OTU abundances (Table 3). These included Sva0071 (1.47–2.06%) and JTB255 (1.47–2.06%), both of which are widespread, and have been implicated in sulfide/sulfur oxidation and dark chemoautotrophic CO_2_ fixation (Ravenschlag et al., 1999; Nercessian et al., 2005; Zheng et al., 2014; Mußmann et al., 2017; Probandt et al., 2018). A third abundant group, HOC36 (0.61–2.01%), is also widely distributed, but poorly known (Probandt et al., 2018). Taken together the results suggest that sulfide and sulfur oxidizers account for a large portion of Gammaproteobacteria diversity in Cod Cove sediments, where they likely complement similarly diverse Deltaproteobacteria in an active sulfur cycle.

### Alphaproteobacteria Distribution

Members of the genus, *Boseongicola* (Rhodobacteriaceae, heterotrophic), dominated the Alphaproteobacteria (6.6% of Alphaproteobacteria), which were generally much less abundant than Delta- or Gammaproteobacteria (Figure 1 and Table 1). *Boseongicola* was originally isolated from an intertidal site at Jeju Island (South Korea; Park et al., 2014), but has since been observed in sediments from depths >3000 m along a latitudinal transect in the Pacific from 0° S to 59°N (Pohlner et al., 2017). Its presence in Cod Cove sediment indicates that it has a potentially cosmopolitan distribution, and that it might be a member of a core community of benthic Alphaproteobacteria (Probandt et al., 2018). Members of *Methyloceanibacter* were also relatively abundant (2.9% of all Alphaproteobacteria). *Methyloceanibacter* includes benthic methylotrophs and methanotrophs (Takeuchi et al., 2014; Vekeman et al., 2016). Methylotrophic representatives likely use methanol and methylamines that have been observed at micromolar concentrations in nearby Lowes Cove intertidal sediments (King et al., 1983; King, 1984), which support a fauna similar to that of Cod Cove. Other relatively abundant Alphaproteobacteria OTUs were affiliated with cultured heterotrophic and sulfur-cycling taxa including *Anderseniella* (Brettar et al., 2007), *Filomicrobium* (Schlesner, 1987), *Pseudahrensia* (Jung et al., 2012), and *Sulfitobacter* (Yoon et al., 2007).

Nonetheless, unclassified and uncultured groups from order to genus levels accounted for a significant fraction of the Alphaproteobacteria OTUs. Two unclassified Rhodobacteraceae were especially notable, since they accounted for 3.2 and 5.2% of total Alphaproteobacteria abundance, respectively. More generally, only 776 (17.9%) of 4344 OTUs were identifiable at the genus level, accounting for only 39.0% of total abundance for the class, outcomes that were comparable to those for Gammaproteobacteria. Collectively these results suggest that Cod Cove harbors a remarkable level of diversity that encompasses taxa that might have limited geographic distributions, as well as cosmopolitan taxa with global distributions. Communities such as these present possibilities for addressing questions about speciation, and micro- to macro-scale determinates of benthic bacterial community assembly.

### Epsilonproteobacteria Distribution

Epsilonproteobacteria were comparable in abundance to Alphaproteobacteria overall (Figure 1 and Table 1), but were represented by far fewer OTUs (353 versus 4344). In contrast to other Proteobacteria groups the Epsilonproteobacteria were largely comprised of OTUs identifiable to genus (96.2%). Two genera dominated bulk, burrow and fecal cast sediment samples: *Sulfurovum* (1.42–6.96%) and *Sulfurimonas* (0.12–0.71%). Both have been reported at similar levels, and were among the most abundant OTUs in Chinese coastal sediments (Zheng et al., 2014). Both oxidize reduced sulfur compounds, and contribute to benthic sulfur cycling (Inagaki et al., 2004; Han and Perner, 2015). A recent proposal to separate Epsilonproteobacteria as a new phylum, Epsilonbacteraeota that includes the Deltaproteobacteria order, Desulfurellales (Waite et al., 2017), does not alter these outcomes. Although the Desulfurallales are anaerobic sulfate reducers that expand the physiological and ecological capacity of the Epsilonbacteraeota, they were comprised of only 96 mostly rare OTUs that collectively accounted for <0.01% of total OTU abundance.

### Bacteroidetes and Chloroflexi Distribution

Numerous additional phyla occur in Cod Cove sediments, but only Bacteroidetes and Chloroflexi consistently accounted for >1–2% of total OTU abundance (Table 1). The Anaerolineae (Chloroflexi) ranged in abundance from 1.9 to 3.0% for sediment samples with no distinct trend among them (Table 1). Most of the Anaerolineae belonged to unclassified lineages (97.9% of total Anaerolineae abundance), but a small number were classified in genera that have been isolated from shallow or deep-sea marine sediments (e.g., *Pelolinea*, Imachi et al., 2014; and *Thermomarinilinea*, Nunoura et al., 2013). Since the class is largely known from non-saline, terrestrial systems, the observation of novel groups in accessible, intertidal sediments offers an opportunity to substantially expand libraries of isolates as well as insights regarding the physiology and ecology of Anaerolineae (Yamada et al., 2006). Contributions to sulfur cycling would be of particular interest, since the class is not currently known to reduce or oxidize sulfur compounds.

Bacteroidetes abundance generally exceeded that of Alphaproteobacteria, accounting for 9.6–14.7% of all OTUs (Table 1). Similar patterns have been reported for other intertidal systems in marked contrast to trends for nearshore bacterioplankton (e.g., Pommier et al., 2007; King et al., 2013). Unclassified or uncultured members of Bacteroidetes group BD2-2, and the classes, Bacteroidia, Cytophagia, and Sphingobacteriia were dominant. Among the Bacteriodetes with cultured representatives, OTUs identified as *Lutimonas* or *Lutibacter* were most abundant. These two genera have been isolated from intertidal sediments as well as several marine invertebrates (Choi and Cho, 2006; Yang et al., 2007; Park et al., 2010, 2015; Kim et al., 2014), and were among the 20 most abundant OTUs in a survey of six distinct intertidal sediments (Zheng et al., 2014).

### Freshly Excreted Gut Sediment Communities

At a class level, the composition of freshly excreted gut sediments was similar to that of other sediment samples, but with several notable exceptions. Deltaproteobacteria, Holophagae, Planctomycetacia, and Sphingobacteriia were distinctly lower in gut sediments, while Epsilonproteobacteria, Gammaproteobacteria and Spirochaetes were distinctly higher (Table 1 and Figure 1). Gammaproteobacteria were especially abundant, accounting for >52% of all OTUs. Major genera included *Colwellia*, *Photobacterium*, *Psychrobacter*, *Pseudoalteromonas*, and *Vibrio*, each of which was typically rare in other sediment samples as well as in sediment-free whole *S. bromophenolosus*.

Relative to surface sediments and fecal casts, the composition of *S*. *bromophenolosus* gut sediments suggests that digestion substantially alters microbial communities. However, very little is known about bacterial fates in enteropneust guts. Carey and Mayer (1990) observed only small decreases in bacterial abundance when comparing *S*. *bromophenolosus* food sources (surficial sediments) with fresh fecal castings. However, they did not examine gut sediment directly, nor consider the possibility of growth within the gut. Dobbs and Guckert (1988) observed differences in the feeding depressions and fecal casts of *Ptychodera bahamensis*, but also did not examine gut contents, and neither group explored changes in specific microbial taxa. Plante and Mayer (1994) explored bacteriolytic activity in the gut of *Stereobalanus canadensis*, but likewise did not conduct phylogenetic analyses of microbial communities.

In contrast, somewhat more is known about the fates of bacteria in molluscs, crustacea and polychaetes. Distinct microbial communities have been documented in the stomachs and guts of *Crassostrea virginica* (King et al., 2012), and *Vibrio* has been documented as a major component of the culturable community of shrimp guts (*Litopenaeus vannamei*, Moss et al., 2000). Plante et al. (1989) observed selective losses of bacteria in the midgut of the polychaete, *Abarenicola vagabunda*, with rapid regrowth in the hindgut. Doubling times of 50–70 min were estimated for taxa that survived mid-gut digestion. Selective losses were also inferred for bacteria digestion by *Arenicola marina*, with bacteriolysis dependent on cell wall structure (e.g., Gram-positive versus Gram-negative) and presence of exopolymeric substances (Plante et al., 1996; Plante and Shriver, 1998). Rapid hindgut regrowth has also been reported for *A*. *marina* (Andresen and Kristensen, 2002).

Similar phenomena occurring in the digestive tract of *S*. *bromophenolosus* could account for selective losses of some taxa and enrichments of others. Blooms of Gammaproteobacteria (e.g., *Colwellia*, *Photobacterium*, *Psychrobacter*, *Pseudoalteromonas*, and *Vibrio*) are of particular interest, since they indicate that *S*. *bromophenolosus* gut sediments might represent “hot spots” that serve as reservoirs for specific taxa at local or even regional scales.

### Sediment-Free Whole *S. bromophenolosus* Communities

The composition of sediment-free, whole animals differed markedly from that of all other samples, including freshly excreted gut sediment (Table 1 and Figure 1). At a class level Bacteriodetes, Deltaproteobacteria, and Gammaproteobacteria were distinctly lower, while Actinobacteria, Alphaproteobacteria, Spartobacteria, and especially Spirochaetes were relatively more abundant. The Spirochaetes were notable, as they accounted for 36% of total OTU abundance. A single unclassified member of the Spirochaetaceae that was most closely related to an anaerobic, haloalkaliphilic isolate from Mono Lake (*Spirochaeta americana*, Hoover et al., 2003) dominated these OTUs.

This same unclassified Spirochaete was present in freshly excreted gut sediments collected independently 2 years earlier, indicating that it might form stable associations with Cod Cove *S. bromophenolosus* populations. Whether this or related Spirochaetes form associations with other *S. bromophenolosus* populations, or other enteropneusts, and what role they might play is unknown. However, many Spirochaetes, including *S*. *americana*, degrade sugars and polysaccharides (Hoover et al., 2003), which suggests that they might be selectively enriched by the copious mucus *S. bromophenolosus* secretes.

Though less abundant than Spirochaete OTUs, a small number of Spartobacteria OTUs most closely related to Chthoniobacterales clone Da101 (Felske et al., 1998) were uniquely associated with sediment-free *S. bromophenolosus*. The functional traits of this group have not yet been identified, but a related soil isolate, *Chthoniobacter flavus* (Sangwan et al., 2004), has been described as a sugar and polysaccharide specialist. Thus, like Spirochaete OTUs, these OTUs might be enriched by *S. bromophenolosus* mucus.

### Community Structure

**FIGURE 2:**
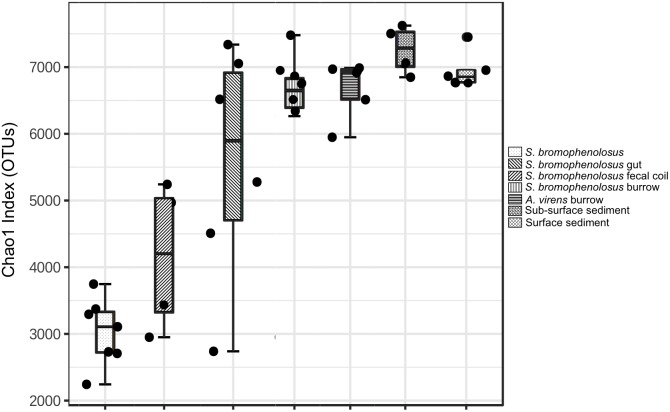
Chao1 index for animal and sediment microbial communities. Chao1 index for whole, sediment-free *S*. *bromophenolosus*, *S*. *bromophenolosus* gut sediments, and various associated sediments; mean, upper and lower quartiles and data extremes indicated. ANOVA: *F* = 18.882, *p* < 0.00001.

To select the most informative OTUs, samples were filtered based on mean abundances and variances prior to analyzing alpha and beta diversity; abundances of the OTUs remaining after filtering were standardized using a centered log ratio transformation. Filtering yielded 2536 informative OTUs from a total of 65,939. The Chao1 richness index derived from the filtered and transformed dataset varied from a low of 3608 ± 391 (standard error, S.E) for whole *S. bromophenolosus* to a maximum of 8709 ± 462 for surface sediment (Figure 2). Analysis of variance (ANOVA) indicated that OTU richness was lowest for whole *S. bromophenolosus* and freshly excreted gut samples, which did not differ from each other, based on a Bonferroni *post hoc* analysis (Supplementary Table 1). Significantly greater richness was observed in surface, sub-surface and burrow wall sediments, which did not differ statistically from each other (Supplementary Table 1).

Richness (Chao1) in Cod Cove surface, sub-surface and burrow wall sediments (Figure 2) fell within a range of values reported for other nearshore marine sediments with similar numbers of observed OTUs (e.g., Wang et al., 2012; Zheng et al., 2014; Choi et al., 2016). Although the database for intertidal systems is limited, it is noteworthy that the lowest richness has been observed for the northernmost sites, e.g., Cod Cove at 44°N (Chao1 = 7840–8709) and Liaodong Bay (China) at 40°N (Chao1 = 4945–6816), both of which experience freezing temperatures and ice scouring in winter (Li et al., 2013) that do not occur at sites on Jeju Island (33°N, Choi et al., 2016) or in Hong Kong (22°N, Wang et al., 2012), which supported higher richness (Chao1 = 8538–14,439). Though one might propose that differences in temperature regimes and physical disturbances account for differences in richness, this relationship has not yet been validated generally, and other variables, e.g., organic matter, benthic primary production, and sediment texture, might prove more important.

The fact that richness did not vary significantly among surface, sub-surface and the two burrow wall sediment types suggests that the impacts of animal populations on microbial communities in Cod Cove might be limited to composition. This could reflect relatively rapid sediment turnover (or mixing) due to bioturbation, and commercial worm and clam harvesting (Brown and Wilson, 1997). Based on cell counts and assays of metabolic potential, Plante and Wilde (2001) also speculated that due to rapid recovery rates, deposit feeders were unlikely to affect benthic microbial community structure. In addition, intrinsic differences between *S. bromophenolosus* and *A*. *virens* might be less important than other variables, e.g., organic contents, in controlling burrow wall community richness.

Relatively low richness in gut sediments (Chao1 was 56% of surface sediment values, Figure 2), along with differences in composition, likely reflects selective losses of taxa and differential population regrowth during sediment passage through the gut. Both phenomena have been observed for polychaetes (Plante et al., 1989; Plante and Shriver, 1998; Andresen and Kristensen, 2002), but neither has been specifically associated with changes in diversity metrics. Although few other studies of marine invertebrates are available for additional comparisons, analyses of stomach and gut microbiomes of oysters (*Crassostrea virginica*) revealed lower values for Chao1 in stomachs. The patterns for oyster stomach and gut richness are consistent with a decrease in diversity during the initial phase of digestion, and an increase in diversity during later stages (King et al., 2012).

Sediment-free, whole *S. bromophenolosus* richness was markedly lower than that of gut sediment or other samples (Figure 2). In addition, many of the low abundance OTUs might have been incidentally associated with the animals’ mucus coating, thus further reducing the number of OTUs that might be considered characteristic of *S. bromophenolosus*. Thus, whether or not *S. bromophenolosus* harbors a core bacterial community or host-specific symbionts remains unknown, although the relatively high abundance of a Spirochete found only in association with whole animals or gut sediments suggests a potentially specific relationship that warrants further exploration.

In contrast to estimates of richness, the Shannon index did not vary statistically among samples with the exception of gut sediments, for which values were lower than all other sample groups (*p* < 0.0001; Supplementary Figure 2 and Supplementary Table 2). This outcome provides additional support for the proposal by Plante and Wilde (2001; see above). The decrease for gut sediments again indicates that *S. bromophenolosus* digestive processes alter microbial diversity. However, those impacts appear short lived, since the Shannon index of fecal castings was equivalent to that of surface sediments. Setting aside the gut sediments, the Shannon indices of the remaining samples were similar to values reported in other studies of nearshore and intertidal systems (e.g., Wang et al., 2012; Zheng et al., 2014; Choi et al., 2016). Prior studies with polychaete and shrimp burrows have also reported little difference in Shannon indices relative to surface and sub-surface sediments (Laverock et al., 2010; Pischedda et al., 2011).

**FIGURE 3:**
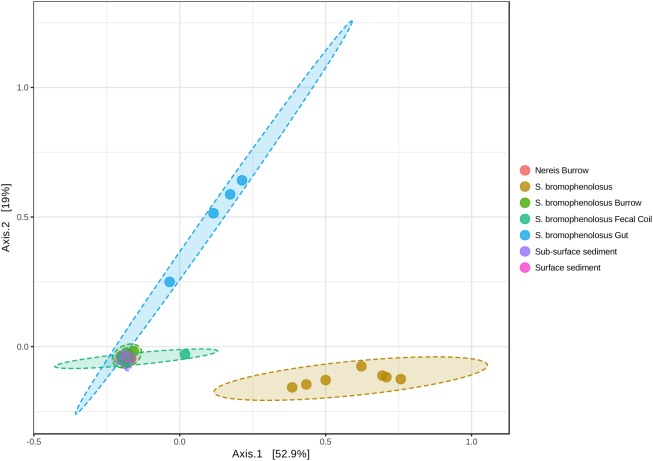
Principal coordinates analysis of animal and sediment microbial communities. Principal coordinates analysis of sample OTUs based on weighted UniFrac distances. PERMANOVA: *r*^2^ = 0.738, *p* < 0.001; PERMDISP: *F* = 2.291, *p* = 0.061.

Principal coordinate analyses based on Bray-Curtis and weighted Unifrac distances yielded sample clusters that were consistent with patterns evident in Chao1 and Shannon indices (Figure 3). Freshly excreted gut sediments and sediment-free whole animals formed distinct clusters, while the remaining sediment samples formed a third group (Figure 3). Differences in the locations of centroids for gut sediment, whole animal, and the several sediment groups were statistically significant based on PERMANOVA (*r*^2^ = 0.694 and 0.706 for Bray-Curtis and weighted Unifrac PCoA, respectively, *p* < 0.001 for both).

**FIGURE 4:**
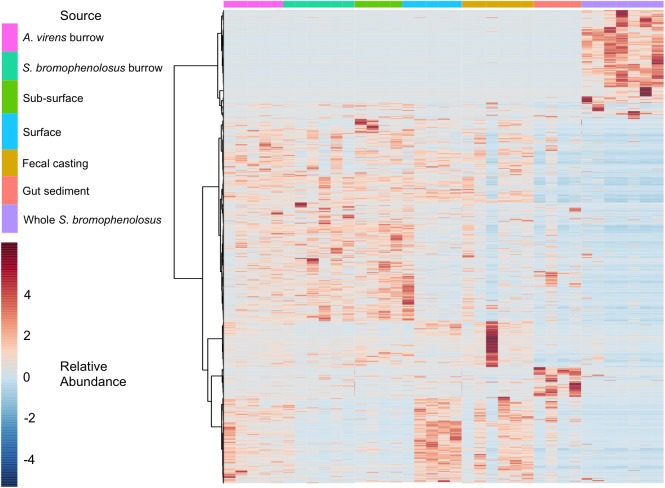
Heatmap of animal and sediment microbial communities. Heatmap of OTU relative abundances observed for individual *S*. *bromophenolosus*, gut and sediment samples. Pearson’s correlation coefficient was used as a distance metric, with clustering based on Ward’s algorithm as implemented by MicrobiomeAnalyst (Dhariwal et al., 2017).

Hierarchical cluster analyses (Supplementary Figure 3A) and heatmaps of OTU abundance (Figure 4) revealed similar relationships for gut sediment and whole animals, but they also showed that burrow and sub-surface sediments largely clustered together, as did fecal casting and surface sediments; in addition, surface and sub-surface sediments clustered distinctly (Supplementary Figure 3B). The associations of burrow wall with sub-surface sediments reflect the fact that burrow wall microbes originate in part from the sub-surface. Many of these populations may vary only modestly in their abundances relative to bulk sediment, in spite of changing biogeochemical conditions within burrows. The depth interval collected during burrow wall sampling, 2–3 mm, might also have included sediment unaffected by the burrows, thus reducing distinctions between burrows and the sub-surface that might be evident with finer resolution sampling.

Although freshly excreted gut sediments harbor distinct microbial communities, that signature appears to be lost within 24 h after deposition on the sediment surface. Four of six fecal casts clustered with sediment surface samples, indicating that their communities had changed substantially from those in the gut. This is consistent with observations by Plante and Wilde (2001), who observed rapid recovery (2 h) of bacterial numbers and Biolog substrate utilization patterns for fecal casts of *B*. *aurantiacus* and *Nereis* (*Alitta*) *succinea*. In this context, it is notable that one of the fecal casts clustered with gut sediments (Supplementary Figure 3B, also evident in Figure 4), possibly indicating that it had been deposited very recently.

Hierarchical cluster analyses indicated that some of the *S*. *bromophenolosus* burrow wall samples were distinct from those of *A*. *virens* burrows (Supplementary Figure 3B), which suggests that the impacts of *S. bromophenolosus* haloorganics on microbial communities might be variable, or possibly constrained to a burrow wall depth interval less than that sampled for this study (e.g., ≤ 2 mm). This outcome agrees in general with observations of Steward et al. (1996), who observed that differences in burrow walls of *B*. *aurantiacus* and surrounding sediments varied with PLFA classes.

Biomarker analysis using linear discriminate analysis effect size (LEfSe, Segata et al., 2011) as implemented in mothur (Kozich et al., 2013) revealed 7195 discriminant features with an LDA score >2 among 65,939 OTUs overall. The top 10 discriminant OTUs for each sample were consistent with patterns in sample taxonomic composition. For example, sediment-free *Saccoglossus* were distinguished by the presence of Spirochaetes, several modestly abundant Acidobacteria, and two Cyanobacteria/chloroplast OTUs that were absent or very low in other samples. Freshly excreted gut sediments were distinguished by abundant OTUs that represented *Pseudoalteromonas*, Vibrionaceae, *Psychrobacter*, and Gracilibacteria, among others (Supplementary Table 3). *S*. *bromophenolosus* and *A*. *virens* burrows were distinguished by members of *Desulfobulbus*, *Sulfurovorum*, and Thiotrichaceae in the former and various uncultured or unclassified Gammaproteobacteria, including the JTB255 marine benthic group, in the latter (Supplementary Table 3).

**FIGURE 5:**
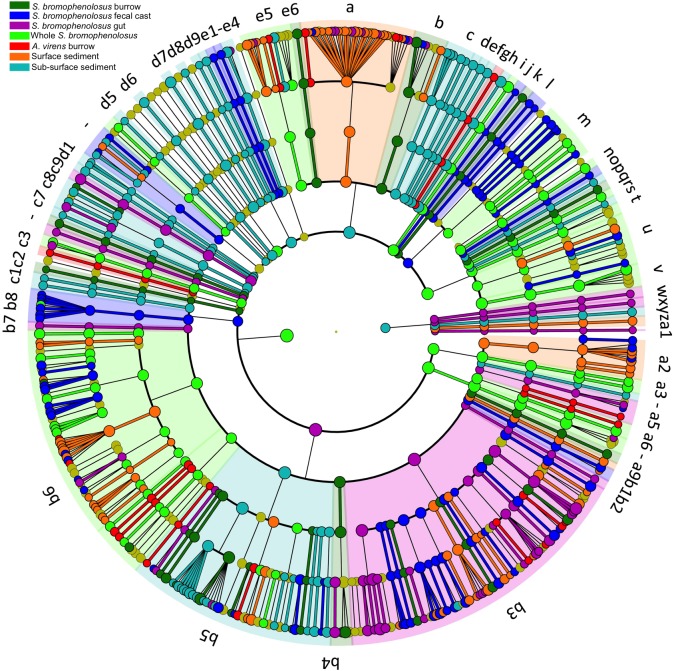
LEfSe cladogram of animal and sediment microbial communities. Cladogram based on LEfSe analysis of the 1000 most abundant OTUs in the data set, showing the phylogenetic distribution of lineages associated with various animal and sediment groups as indicated by the source color key for lineages with LDA values ≥3.5. Circle diameters are proportional to OTU abundance with phylogenetic levels from domain to family from outside to inside. Key to discriminant lineages a, Flavobacteriia; b, Cytophagia; c, Bacteroidia; d, Bacteroidetes_vadinHA17; e, Bacteroidetes_unclassified; f, Bacteroidetes_VC2_1_Bac22; g, Bacteroidetes_Incertae_Sedis; h, Bacteroidetes_BD2_2; i, Bacteria_unclassified; j, Atribacteria; k, Aminicenantes; l, Thermoleophilia; m, Actinobacteria; n, Subgroup_6; o, Subgroup_26; p, Subgroup_22; q, Subgroup_21; r, Subgroup_2; s, Subgroup_18; t, Solibacteres; u, Holophagae; v, Acidobacteria; w, Lokiarchaeota; x, Thermoplasmata; y, Bathyarchaeota; z, Archaea_unclassified; a1, Ancient_Archaeal_Group_AAG; a2, Verrucomicrobiae; a3, Spartobacteria; a4, R76_B128; a5, Opitutae; a6, OPB35_soil_group; a7, Spirochaetes; a8, Zetaproteobacteria; a9, Proteobacteria_unclassified; b1, Proteobacteria_Incertae_Sedis; b2, Milano_WF1B_44; b3, Gammaproteobacteria; b4, Epsilonproteobacteria; b5, Deltaproteobacteria; b6, Betaproteobacteria; b7, Alphaproteobacteria; b8, AEGEAN_245; b9, Planctomycetacia; c, Bacteroidia; c1, Phycisphaerae; c2, Peregrinibacteria; c3, Candidatus_Moranbacteria; c4, Nitrospira; c5, Marinimicrobia__SAR406ade_; c6, Latescibacteria; c7, KSB3__Modulibacteria_; c8, Ignavibacteria; c9, Gracilibacteria; d1, BD2_11_terrestrial_group; d2, Fusobacteriia; d3, Clostridia; d4, Bacilli; d5, Fibrobacteria; d6, Deferribacteres_Incertae_Sedis; d7, Chloroplast; d8, SJA_15; d9, Dehalococcoidia; e1, Chloroflexi_unclassified; e2, Caldilineae; e3, Ardenticatenia; e4, Anaerolineae; e5, Sphingobacteriia; e6, SB_5.

The 1000 most abundant OTUs, which comprised 82.0% of all reads, were also analyzed by LEfSe using the Huttenhower Galaxy platform (Segata et al., 2011). Discriminant OTUs were derived primarily from Gamma- and Deltaproteobacteria for sediment groups, and from a mix of phyla and classes, including Spirochaetes, for whole *S*. *bromophenolosus* (Figure 5).

## Conclusion

Results from this study indicated that sediment-free whole *S. bromophenolosus* harbors a Spirochaete OTU and a small number of additional taxa that might constitute a core microbiome or represent symbionts, the functions of which remain unknown at present. The Spirochaete OTU was also observed in *S. bromophenolosus* gut sediments, but not other sediment samples, which further supports a possible previously unrecognized host-specific association. Gut sediments also harbored Gammaproteobacteria that were uncommon in other sediment samples, and that might have “bloomed” within *S. bromophenolosus* after the initial stages of digestion. However, the relative abundance of many other taxa was substantially reduced in gut sediments, indicating differential losses during early stages of digestion. Nonetheless, the composition and diversity of *S. bromophenolosus* fecal castings resembled that of surface sediments, and was largely distinct from gut sediments, which indicated that recovery was rapid post-deposition.

Although burrow wall, surface and sub-surface samples shared most of the sediment OTUs, distinct communities developed within each as indicated by hierarchical cluster and LEfSe analyses. The observed differences likely reflected changes in the relative abundance of OTUs due to local differences in variables such as oxygen availability, benthic primary production, and organic matter availability. For example, surface sediments are continuously oxygenated, sub-surface sediments are anoxic, and burrow wall sediments are oxygenated variably. At a large scale, i.e., the mudflat, sediment mixing through bioturbation, physical processes and anthropogenic activity might act to homogenize community composition. Although Cod Cove, Maine is a relatively isolated intertidal system on a marine river, community members included groups with cosmopolitan distributions and roles in sulfur cycling, e.g., Gammaproteobacteria BD7 and Sva0071, as well as numerous novel OTUs representing a large number of phyla.

## Ethics Statement

This study is exempt from animal use requirements as none exist for benthic marine invertebrates.

## Author Contributions

GK designed the work, collected the samples, analyzed the data, and wrote the manuscript.

## Conflict of Interest Statement

The author declares that the research was conducted in the absence of any commercial or financial relationships that could be construed as a potential conflict of interest.

## Footnotes

1 www.microbiomeanalyst.ca
