# Supplementary material for: Microbiomes of the Enteropneust, Saccoglossus bromophenolosus, and Associated Marine Intertidal Sediments of Cod Cove, Maine
Source: Front Microbiol. 2018 Dec 14;9:3066. doi: 10.3389/fmicb.2018.03066 (PMC6315191; doi:10.3389/fmicb.2018.03066)
Supplement: Supplementary file 1 [file Table_1.DOCX]

| **Sample** | **Chao1** | **Groups** | | |
| --- | --- | --- | --- | --- |
|  |  |  |  |  |
| Surface | 6962 (394) | A |  |  |
| Sub-surface | 7257 (440) | A |  |  |
| *A. virens* burrow | 6671 (394) | A |  |  |
| *S. bromophenolosus* burrow | 6704 (360) | A |  |  |
| *S. bromophenolosus* fecal | 5572 (360) | A | B |  |
| *S. bromophenolosus* gut | 4151 (440) |  | B | C |
| *S. bromophenolosus* | 3029 (332) |  |  | C |

Supplementary Table 1. Chao1 index (mean, standard error in parens) for various sediment and *S. bromophenolosus* samples. Means of samples with the same group letters do not differ significantly based on ANOVA with Bonferroni post-hoc test and a correction for multiple comparisons.

Supplementary Table 2. Shannon index (mean, standard error in parens) for various sediment and *S. bromophenolosus* samples. Means of samples with the same group letters do not differ significantly based on ANOVA with Bonferroni post-hoc test and a correction for multiple comparisons.

| **Sample** | **Shannon** | **Groups** | |
| --- | --- | --- | --- |
|  |  |  |  |
| Surface | 5.95 (0.23) | A |  |
| Sub-surface | 5.87 (0.22) | A |  |
| *A. virens* burrow | 5.88 (0.23) | A |  |
| *S. bromophenolosus* burrow | 5.57 (0.21) | A |  |
| *S. bromophenolosus* fecal | 5.64 (0.21) | A |  |
| *S. bromophenolosus* gut | 4.28 (0.25) |  | B |
| *S. bromophenolosus* | 5.97 (0.19) | A |  |
