# Supplementary material for: Microbiomes of the Enteropneust, Saccoglossus bromophenolosus, and Associated Marine Intertidal Sediments of Cod Cove, Maine
Source: Front Microbiol. 2018 Dec 14;9:3066. doi: 10.3389/fmicb.2018.03066 (PMC6315191; doi:10.3389/fmicb.2018.03066)
Supplement: Supplementary file 2 [file Table_2.DOCX]

Supplementary Table 3. Top 10 discriminant features identified for each sample type by LEfSE analysis as implemented in mothur.
