# Supplementary material for: Microbiomes of the Enteropneust, Saccoglossus bromophenolosus, and Associated Marine Intertidal Sediments of Cod Cove, Maine
Source: Front Microbiol. 2018 Dec 14;9:3066. doi: 10.3389/fmicb.2018.03066 (PMC6315191; doi:10.3389/fmicb.2018.03066)
Supplement: Supplementary file 3 [file Data_Sheet_1.docx]

**Supplementary Figure Legends**

**Supplementary figure 1**. **Non-metric multidimensional scaling analysis of Deltaproteobacteria**. Non-metric multi-dimensional scaling analysis of OTUs assigned to Deltaproteobacteria for whole, sediment-free *S*. *bromophenolosus*, *S*. *bromophenolosus* gut sediments, and various associated sediments. Dissimilarities based on Bray-Curtis distances; Kruskal’s stress = 0.033.

**Supplementary figure 2**. **Shannon index for animal and sediment microbial communities**. Shannon index for whole, sediment-free *S*. *bromophenolosus*, *S*. *bromophenolosus* gut sediments, and various associated sediments; mean, upper and lower quartiles and data extremes indicated. ANOVA: F = 5.995, p < 0.00033.

**Supplementary figure 3a**. **Hierarchical cluster analysis of animal and sediment microbial communities**. Hierarchical cluster analysis of animal and sediment sample OTUs based on the weighted UniFrac metric with Ward’s method for clustering. **3b**. **Hierarchical cluster analysis of sediment microbial communities**. Hierarchical cluster analysis of sediment sample OTUs based on Bray-Curtis dissimilarities with Ward’s method for clustering.
