# Supplementary figures and images for: Microbiomes of the Enteropneust, Saccoglossus bromophenolosus, and Associated Marine Intertidal Sediments of Cod Cove, Maine
Source: Front Microbiol. 2018 Dec 14;9:3066. doi: 10.3389/fmicb.2018.03066 (PMC6315191; doi:10.3389/fmicb.2018.03066)

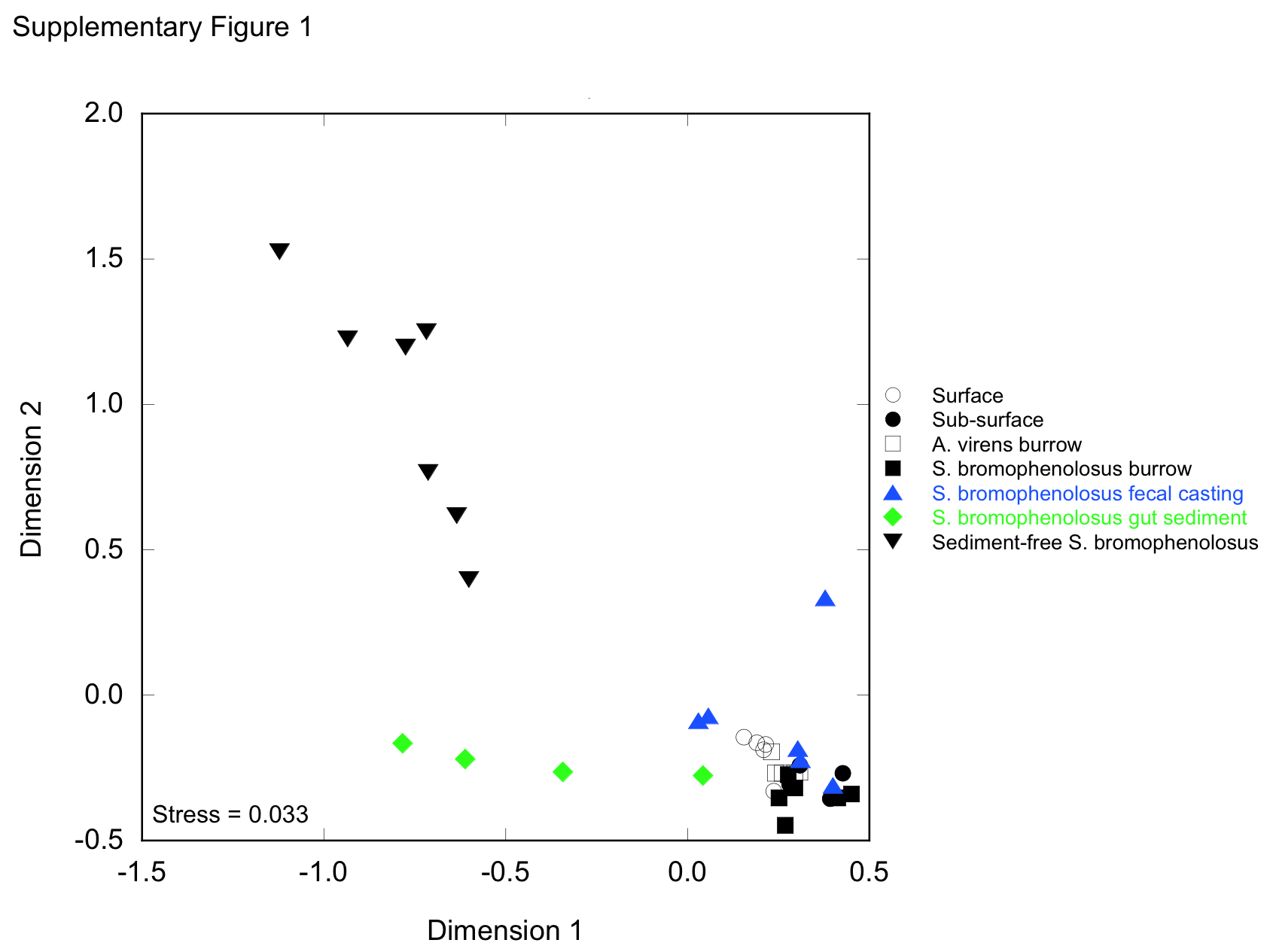

Supplement: Supplementary file 4 [file Image_1.TIFF]

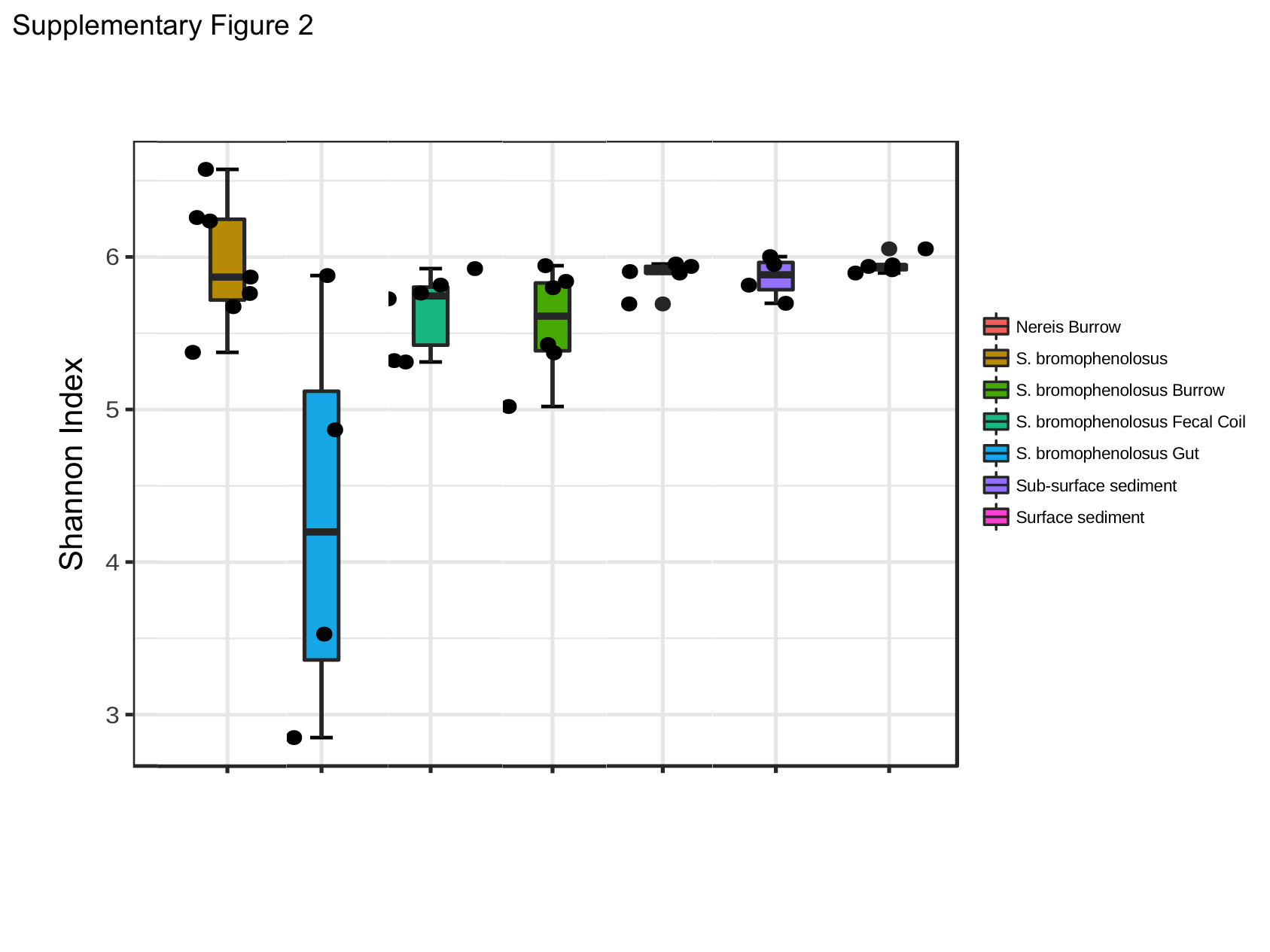

Supplement: Supplementary file 5 [file Image_2.TIFF]

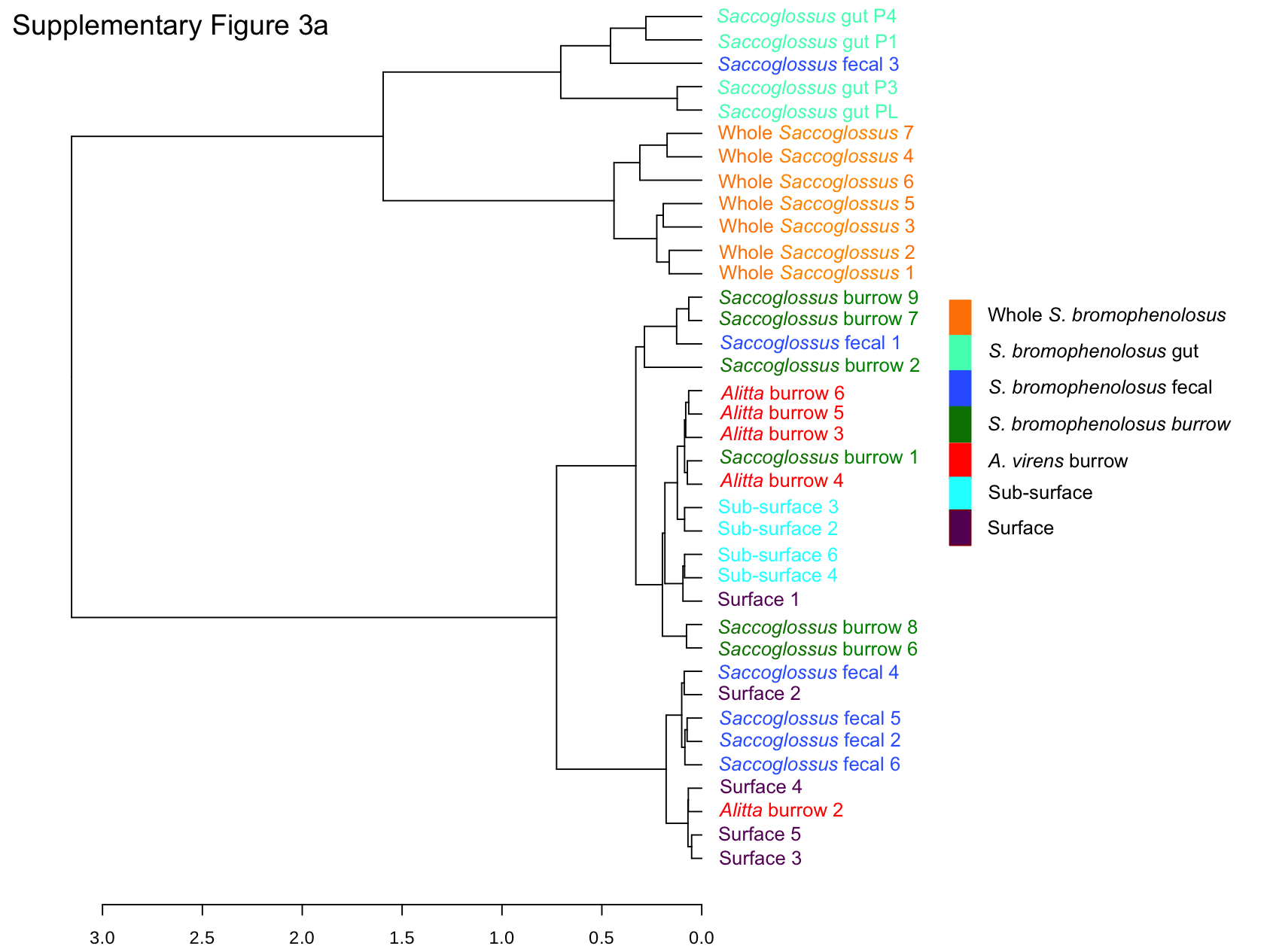

Supplement: Supplementary file 6 [file Image_3.TIFF]

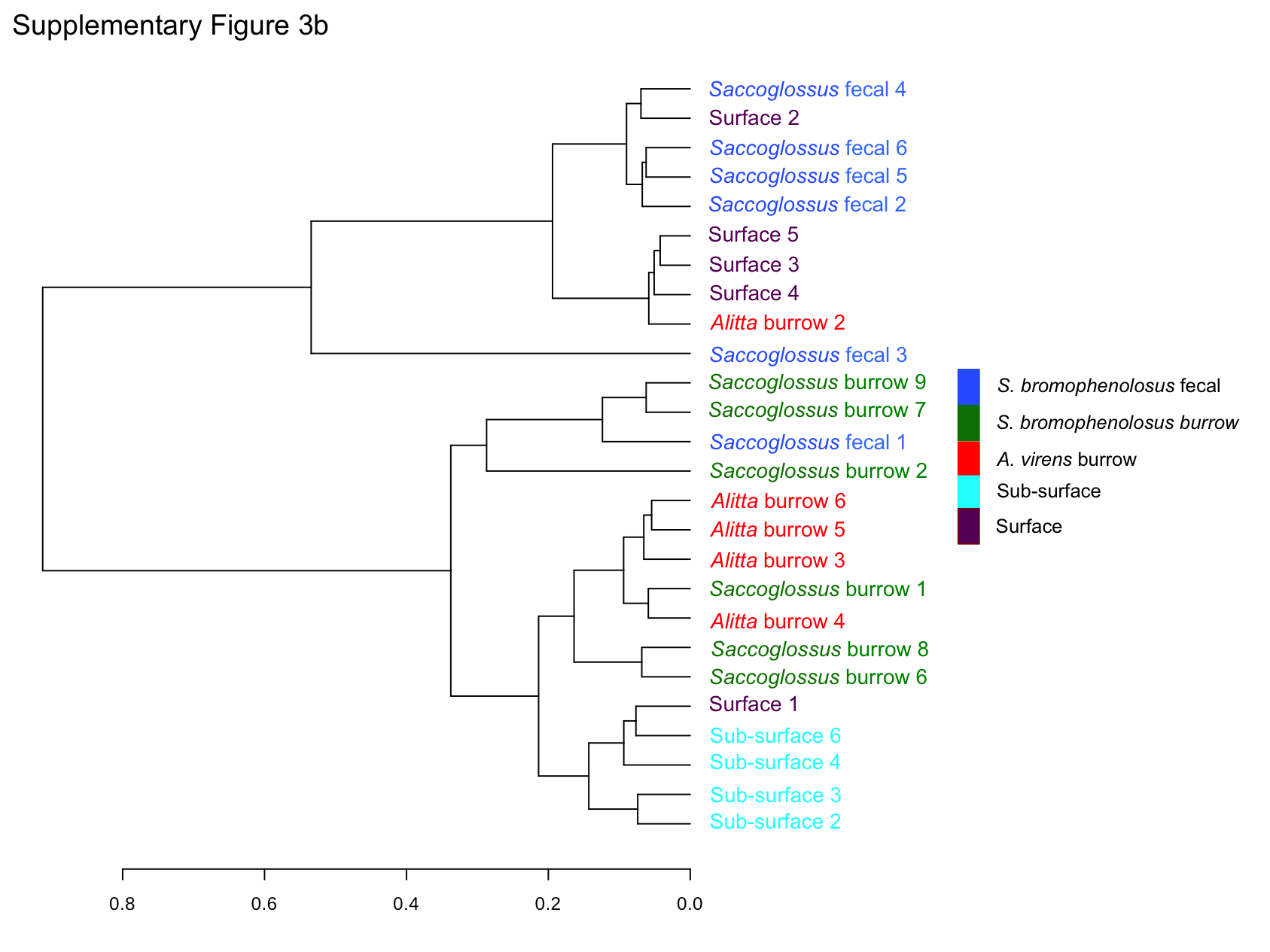

Supplement: Supplementary file 7 [file Image_4.TIFF]
